# Supplementary figures and images for: “Frozen evolution” of an RNA virus suggests accidental release as a potential cause of arbovirus re-emergence
Source: PLoS Biol. 2020 Apr 28;18(4):e3000673. doi: 10.1371/journal.pbio.3000673 (PMC7188197; doi:10.1371/journal.pbio.3000673)

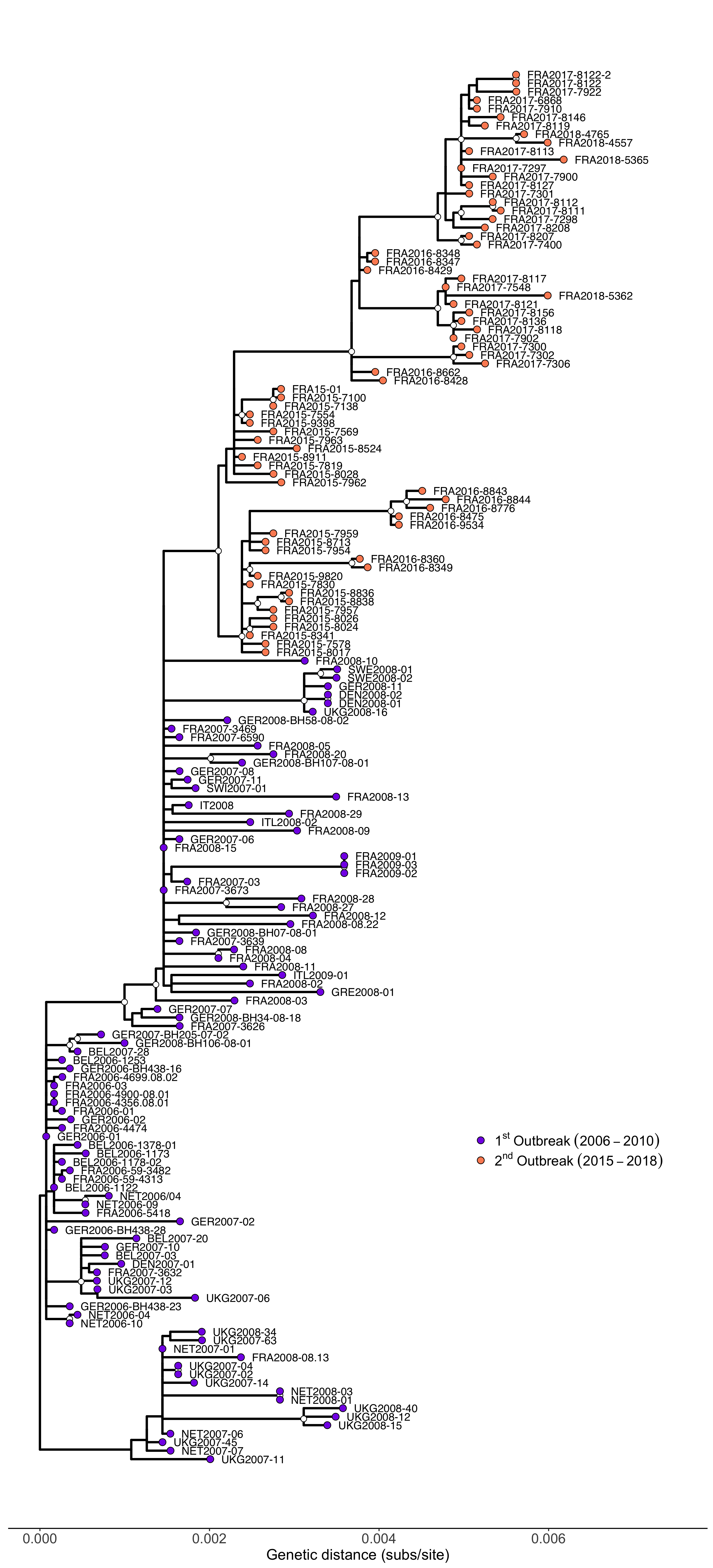

Supplement: S1 Fig — ML tree estimated in PhyML. The scale shows substitutions per site. Clades represented 700 or more times within 1,000 bootstraps are indicated by a white circle. Samples from the first outbreak are shown with purple circles, while samples form the second outbreak are shown with an orange circle. BTV-8, bluetongue virus serotype 8; ML, maximum likelihood. (TIF) [file pbio.3000673.s004.tif]

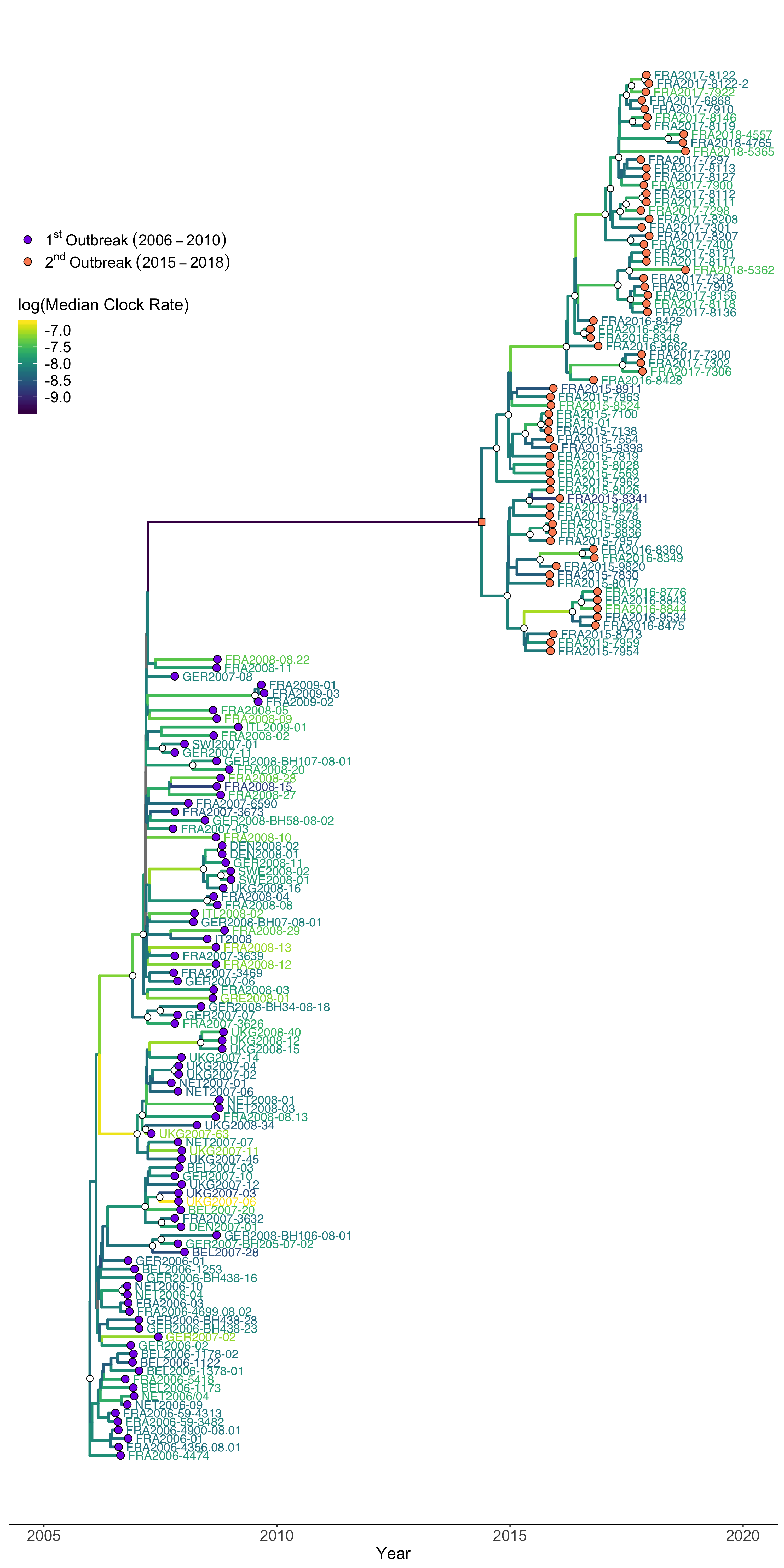

Supplement: S2 Fig — Maximum clade credibility time-calibrated phylogenetic tree generated in BEAST. The tree is scaled in years, with the final sampling date being October 2018. Clades with posterior support of 0.9 or higher are indicated by a white circle. Samples from the first outbreak are shown with a purple circle, while samples from the second are shown with orange circles. The branches are coloured accordingly to their median evolutionary rate across the posterior (see heatmap within the figure). BTV-8, bluetongue virus serotype 8. (TIF) [file pbio.3000673.s005.tif]
